# Identification of intermediate conformations in the photocycle of the light-driven sodium-pumping rhodopsin KR2

**DOI:** 10.1016/j.jbc.2021.100459

**Published:** 2021-02-24

**Authors:** Masaki Tsujimura, Hiroshi Ishikita

**Affiliations:** 1 Department of Applied Chemistry, The University of Tokyo, Bunkyo-ku, Tokyo, Japan; 2 Research Center for Advanced Science and Technology, The University of Tokyo, Meguro-ku, Tokyo, Japan

**Keywords:** rhodopsin, 7-helix ligand-gated channel, proton pump, proton transport, X-ray crystallography, sodium transport, optogenetics, water cluster, low-barrier hydrogen bond, quantum mechanical/molecular mechanical approach, DFT, density functional theory, HOMO, highest occupied molecular orbital, LUMO, lowest unoccupied molecular orbital, PCM, polarizable continuum model, QM/MM, quantum mechanical/molecular mechanical, TR-SFX, time-resolved serial femtosecond crystallography, XFEL, X-ray free electron laser

## Abstract

The light-driven rhodopsin KR2 transports Na^+^*via* the M- and O-states. However, the mechanisms by which the retinal regulates Na^+^ pumping is unknown, in part because KR2 adopts both pentamer and monomer forms in crystal structures and in part because these structures show differences in the protein conformation near the Schiff base, even when they are of the same intermediate state within the photocycle. A particular open question is the nature of the H-bond networks and protonation state in the active site, including Asp116. Here, we analyze the protonation state and the absorption wavelength for each crystal structure, using a quantum mechanical/molecular mechanical approach. In the pentamer ground state, the calculated absorption wavelength reproduces the experimentally measured absorption wavelength (530 nm). The analysis also shows that ionized Asp116 is stabilized by the H-bond donations of both Ser70 and a cluster of water molecules. The absorption wavelength of 400 nm in the M-state can be best reproduced when the two O atoms of Asp116 interact strongly with the Schiff base, as reported in one of the previous monomer ground state structures. The absorption wavelengths calculated for the two Na^+^-incorporated O-state structures are consistent with the measured absorption wavelength (∼600 nm), which suggests that two conformations represent the O-state. These results may provide a key to designing enhanced tools in optogenetics.

Microbial rhodopsins, which are involved in light-dependent biological functions in microorganisms, contain a retinal Schiff base as a chromophore (1, 2). The driving force of the Na^+^-pumping rhodopsin KR2 from *Krokinobacter eikastus* is provided by photoisomerization of the all-*trans* retinal chromophore, which is covalently attached to Lys255 *via* the protonated Schiff base, to 13-*cis* (3). KR2 can also transport K^+^ following mutations in Asn61 (4), Arg109 (5), Ser254 (6), and Gly263 (4, 6, 7). Because the transport of Na^+^ and K^+^ plays a role in neural activity, KR2 is considered a potential tool for use in optogenetics (8, 9).

Microbial rhodopsins have one or two charged groups (counterions) at conserved positions near the Schiff base (*e.g.*, Asp85, Thr89, and Asp212 in bacteriorhodopsin, BR). Asn112 and Asp116 in KR2 structurally correspond to Asp85 and Thr89 in BR, respectively (Fig. 1*A*). The difference in the acidic residue position with respect to the Schiff base is responsible for the difference in the absorption wavelengths of BR (568 nm (10)) and KR2 (530 nm (11)) (12). Asp251 in KR2 is conserved as Asp212 in BR (Fig. 1*A*).

**Figure 1:**
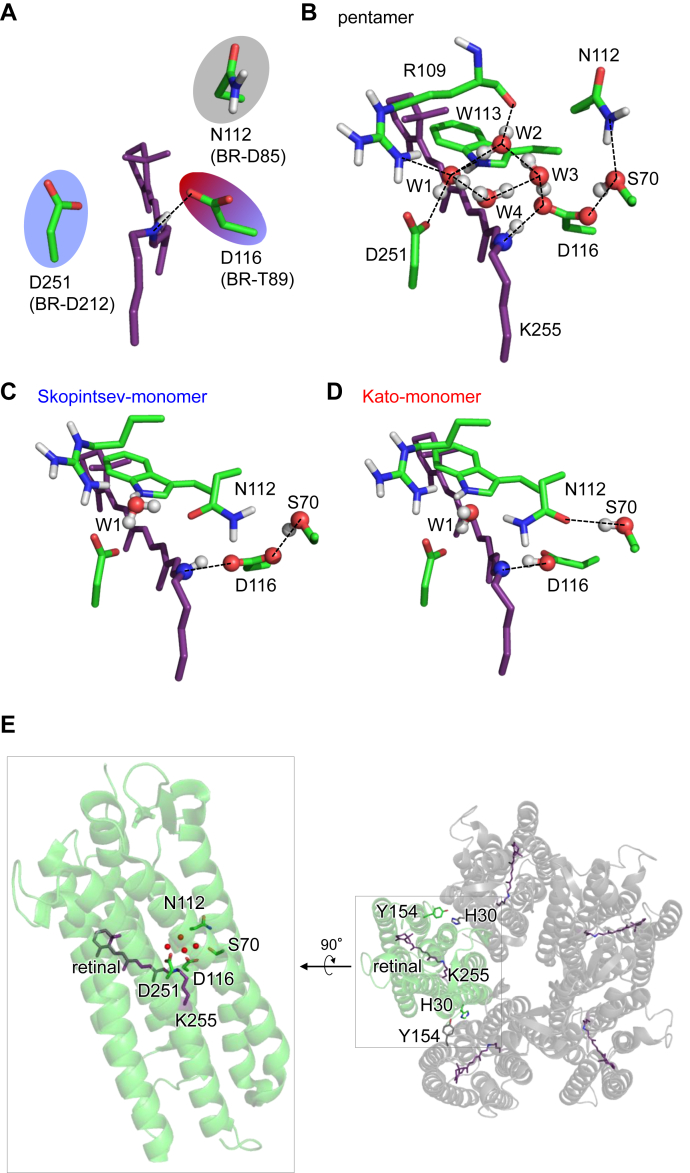
**Ground state structures.***A*, arrangement of the counterions (11). *Gray*, *sky blue*, and *red circles* indicate uncharged, distal charged, and proximal charged residues, respectively. *B–D*, the H-bond network of the Schiff base in the QM/MM-optimized KR2 ground state structures. *B*, Pentamer structure (11) with a cluster of water molecules W1 (HOH-434), W2 (HOH-501), W3 (HOH-437), and W4 (HOH-512). *C*, Skopintsev monomer structure (15). *D*, Kato monomer structure (4). *Dotted lines* indicate H-bonds. *E*, overview of the monomer unit in the pentameric assembly.

KR2 exists as a pentamer in crystals at physiological pH (6, 11), detergent micelles (6), and lipid membranes (13, 14). When the pentameric assembly is obstructed by mutations at the oligomerization interface (*e.g.*, H30L, H30K, and Y154F), the Na^+^ transport activity is inhibited (6). Thus, the pentameric form may be functionally more relevant to KR2 than the monomeric form (6). Tyr154, which is oriented away from the *β*-ionone ring moiety of the retinal Schiff base, forms an H-bond with His30 in the adjacent KR2 monomer unit (Fig. 1*E*).

The pentameric (11) and monomeric (4, 15) KR2 structures show remarkable structural differences, specifically at the Schiff base moiety. In all ground state structures, the Schiff base forms an H-bond with Asp116, while a water molecule (W1) forms an H-bond with Arg109 and Asp251 (Fig. 1, *B*–*D*). The Asp116…Ser70 H-bond is present in the pentamer structure (11) (Fig. 1*B*), as well as the monomer structure reported by Skopintsev *et al*. (15) (Skopintsev monomer structure, Fig. 1*C*), whereas it is absent in the monomer structure reported by Kato *et al*. (4) (Kato monomer structure, Fig. 1*D*). A cluster of four water molecules (W1–W4, Fig. 1*B*) exists at the Schiff base moiety in the pentamer structure (11), whereas the corresponding cluster does not exist in the two monomer structures (4, 15) because of the presence of the Asn112 side chain (Fig. 1, *C* and *D*). However, how the structural difference at the Schiff base moiety between the pentameric and monomeric forms affects the KR2 function remains unclear.

The mechanism underlying Na^+^ pumping through the positively charged Schiff base moiety in KR2 is an open question. The Schiff base forms an H-bond with Asp116 during the photocycle, as suggested in studies of X-ray crystallography (4, 6, 7, 11, 15), Fourier-transform infrared (FTIR) spectroscopy (16), and resonance Raman spectroscopy (17). As a significant shift of 32 nm in the absorption wavelength was observed upon the D116N mutation, Asp116 is considered to be deprotonated in the ground state (4). The proton migrates from the Schiff base to Asp116 upon M-state formation (17). The proton returns to the Schiff base (17) and the uptake of Na^+^ occurs (3) upon O-state formation (M-state decay).

When Asp116 is protonated at low pH in the ground state, the Asp116 side chain orients away from the Schiff base and forms an H-bond with Ser70 and Asn112 (4). The Asp116 conformation in the low-pH ground state may resemble that in the M-state structure because Asp116 is protonated in the M-state (17). Based on these, it was previously proposed that Asp116, which is deprotonated in the ground state, accepts the proton and orients away from the Schiff base in the M state, thus triggering the deprotonated Schiff base to accept Na^+^, proceed to the O-state formation, and conduct Na^+^ (4, 9, 18).

The recent X-ray diffraction (XRD) structure of the O-state in the pentameric form (11) (XRD O-state structure) shows that the Na^+^-binding site does not involve the Schiff base (Fig. 2*A*). This suggests that the proposed movement of the Asp116 side chain away from the Schiff base (4, 9, 18) may not necessarily be required for formation of the M-state. However, the Na^+^-binding site in the XRD O-state structure differs from that in the O-state structure obtained from time-resolved serial femtosecond crystallography (TR-SFX) using an X-ray free electron laser (XFEL) (XFEL O-state structure) (15). The Na^+^-binding site is formed by the side chains of Asp116, Ser70, and Asn112 and the backbone O of Val67 in the XRD O-state structure (11) (Fig. 2*A*), whereas it is formed by the side chains of Asn112 and Asp251 in the XFEL O-state structure (15) (Fig. 2*B*). It should also be noted that two water molecules at the Schiff base moiety of the pentamer ground state structure (Fig. 1*B*) appear to have moved to the positions at the Val66, Ser70, Ile115, and Asp116 moieties in the XRD O-state structure (11) (W2' and W3', Fig. 2*A*).

**Figure 2:**
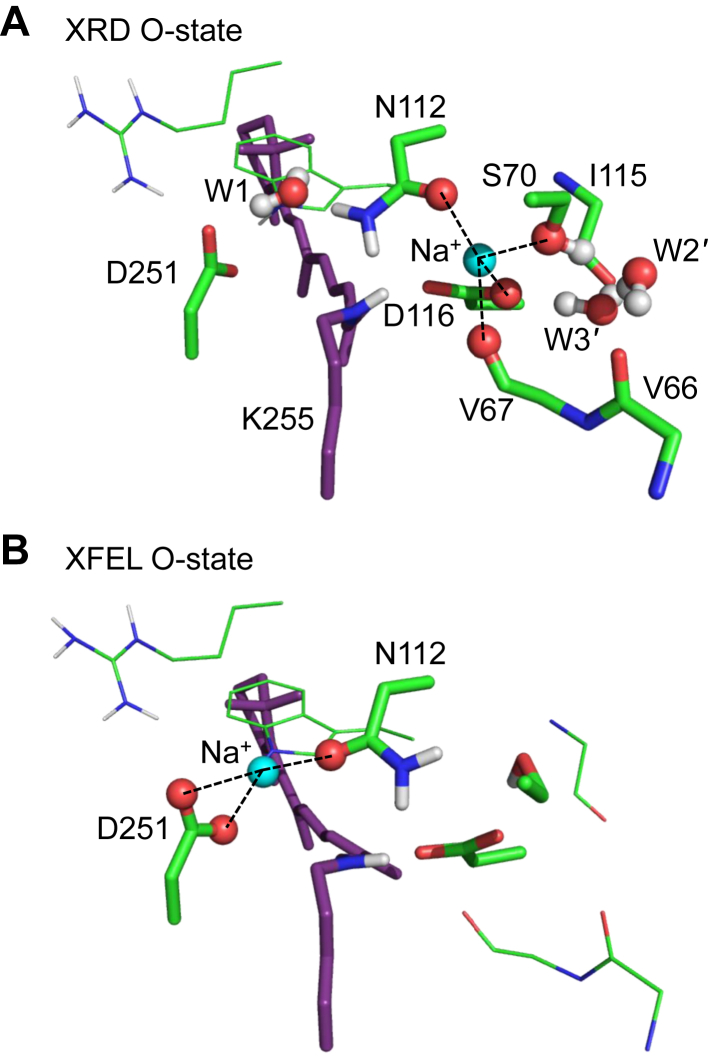
**O-state structures.***A*, XRD O-state structure (11). *B*, XFEL O-state structure (15). *Dotted lines* indicate interactions between Na^+^ and O sites.

In microbial rhodopsins, protein function and absorption wavelengths are predominantly determined by the difference in the protein electrostatic environment at the retinal Schiff base moiety (12). Notably, the small difference in the polar residues at the retinal Schiff base binding site is responsible for the difference in the absorption wavelength among microbial rhodopsins with similar functions (12), which is also the case for the difference in the protein conformation at the retinal binding site for each intermediate state (*e.g.*, Fig. 1 for the ground state) among the reported KR2 crystal structures (4, 11, 15). To clarify the involvement of each protein conformation in the functionally relevant intermediate state in the ground → M → O-state transition, we analyze how each KR2 intermediate conformation affects proton transfer between the Schiff base and Asp116 and the absorption wavelength of the retinal Schiff base, using a quantum mechanical/molecular mechanical (QM/MM) approach.

## Results

### Protonated Schiff base in the ground state

**Figure 3:**
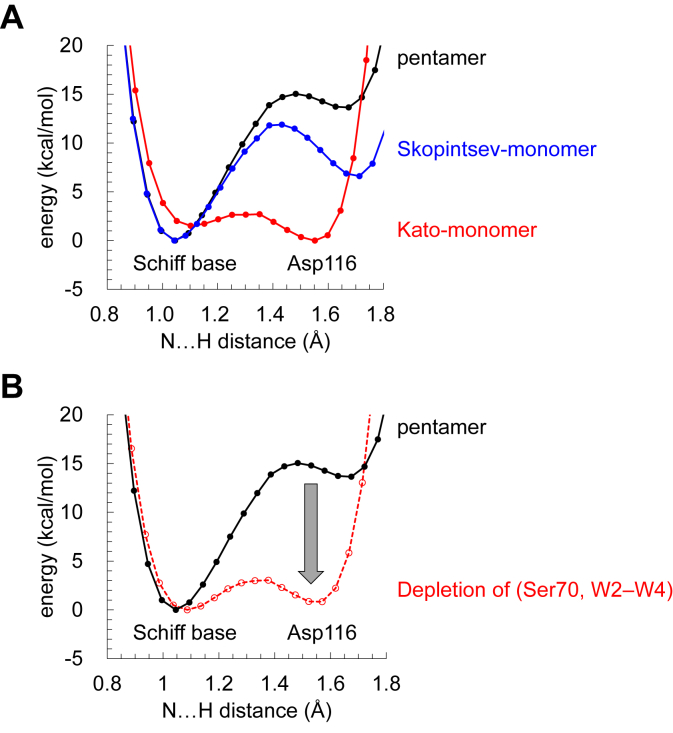
**Potential energy profiles of the H-bond between the Schiff base and Asp116.***A*, three ground-state structures. *B*, changes in the potential energy profile of the pentamer structure (*black solid curve*) in response to the W2–W4 removal and the S70G mutation (*red dotted curve*). The *gray arrow* indicates the shift in p*K*_a_(Asp116) with respect to p*K*_a_(Schiff base).

The difference in the H-bond pattern observed in the three ground state structures (Fig. 1, *B*–*D*) implies a difference in the p*K*_a_ value of the retinal Schiff base. Consistently, the potential energy profile of the H-bond between the Schiff base and Asp116 indicates that p*K*_a_(Schiff base) > p*K*_a_(Asp116) (*i.e.*, the proton is predominantly localized at the Schiff base moiety) in the pentamer and Skopintsev monomer structures, whereas p*K*_a_(Schiff base) ≈ p*K*_a_(Asp116) (*i.e.*, the proton is exchangeable between the two moieties) in the Kato monomer structure (Figs. 3*A* and S1).

In particular, the pentamer/Kato monomer structures show a significant difference in (i) the presence/absence of W2–W4 and (ii) the side-chain orientation of Asn112, serving as an H-bond donor/acceptor of Ser70 (Fig. 1, *B* and *D*). Asn112 serves as an H-bond acceptor of Ser70 in the Kato monomer structure (Fig. 1*D*). In contrast, Asn112 serves as an H-bond donor to Ser70 in the pentamer structure; thus, Ser70 donates an H-bond to Asp116, which decreases p*K*_a_(Asp116) (Fig. 1*B*).

Removal of W2–W4 and mutation of Ser70 to glycine make the shape of the potential energy profile for the pentamer structure symmetric, *i.e.*, p*K*_a_(Schiff base) ≈ p*K*_a_(Asp116) (Fig. 3*B*), which resembles that for the Kato monomer structure (Fig. 3*A*). Indeed, the difference in |p*K*_a_(Schiff base) – p*K*_a_(Asp116)| between the two KR2 structures can be explained by the electrostatic contributions of W2–W4 and the Ser70 side chain (Table 1). These results suggest that (i) the presence of W2–W4 and (ii) the H-bond donation from Ser70, which is due to the H-bond donation from Asn112, stabilize the ionized state of Asp116, leading to p*K*_a_(Schiff base) > p*K*_a_(Asp116) in the ground state pentamer structure.

**Table 1:** Contributions of the key components to the energy difference between the protonated Schiff base and protonated Asp116 (Δ*E*) in the pentamer structure (kcal/mol)

Components	Δ*E*	Contributions to Δ*E*
Pentamer ground state	13.6^a^	
Ser70^b^		+6.1
W2–W4^c^		+6.7
Total		+12.8

a See Figure 3.

b Obtained from the S70G mutant structure.

c Obtained from the W2–W4-depleted structure.

### Deprotonated/protonated Schiff base in M-/O-states

**Figure 4:**
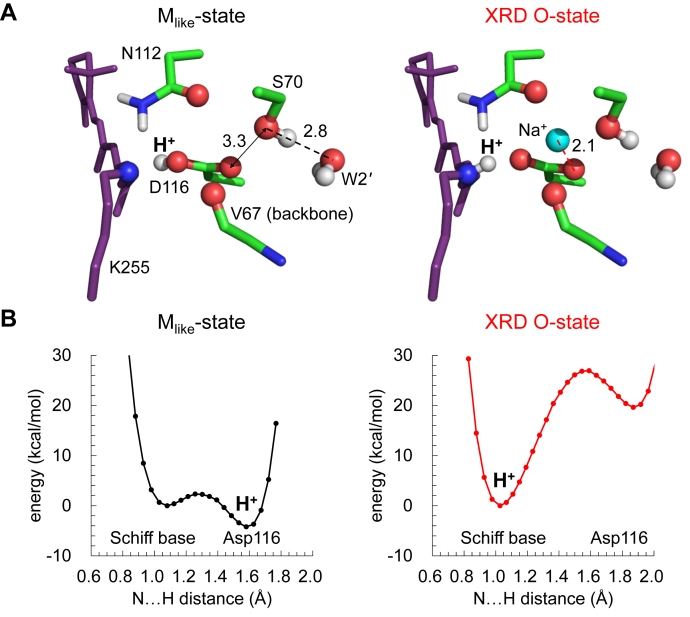
**M_like_- and O-state structures.***A*, Schiff base moiety in the QM/MM-optimized M_like_- and XRD O-state structures. Distances with Ser70/Na^+^ are shown in Å. *Dotted lines* indicate interactions with Ser70/Na^+^. *B*, potential energy profiles of the H-bond between the Schiff base and Asp116 in the M_like_- and XRD O-state structures.

As an M-state structure, in which Na^+^ is not incorporated into the binding site at the Asp116 moiety in the O-state structure, has not been reported, we tentatively use the Na^+^-depleted XRD O-state structure as an M state structure (M_like_-state structure, see SI for the QM/MM-optimized atomic coordinates). QM/MM calculations show that p*K*_a_(Schiff base) < p*K*_a_(Asp116) in the M_like_-state structure, in which Na^+^ does not exist at the binding site (Fig. 4*B*). In contrast, p*K*_a_(Schiff base) > p*K*_a_(Asp116) as Na^+^ is incorporated into the binding site in the XRD O-state structure (Fig. 4*B*). In the M_like_-state structure, W2–W4 are absent and Ser70 donates an H-bond not to Asp116, but instead to W2' (Fig. 4*A*), which increases p*K*_a_(Asp116) (Fig. 4*B*).

In contrast, in the XRD O-state structure, Asp116 interacts with positively charged Na^+^, which decreases p*K*_a_(Asp116) (Fig. 4, *A* and *B*). It seems likely that the proton at the Asp116 moiety migrates again toward the Schiff base moiety in the O-state structure, as Na^+^ is incorporated into the binding site.

### Absorption wavelengths calculated using the protein structures

*Ground state*. The calculated absorption wavelength is 540 nm in the pentamer ground state structure (Table 2), which is consistent with the experimentally measured wavelength of 530 nm (11). The calculated absorption wavelength of the Skopintsev monomer ground state structure is 35 nm shorter than that of the pentamer ground state structure (and the experimentally measured value, Table 2). The difference in the conformation of Asn112 (Fig. 1, *B* and *C*) differentiates the absorption wavelengths of the pentamer and Skopintsev monomer structures by 11 nm. In addition, the experimentally measured absorption wavelength of ∼530 nm (3, 4, 11, 19) was obtained at a physiological pH, where KR2 exists as a pentamer. Thus, the slightly short absorption wavelength for the Skopintsev monomer ground state structure may also be due to the discrepancy in pH.

**Table 2:** Calculated and experimentally measured wavelengths (nm)

State	Structure	Calculated	Measured
Ground state	Pentamer^a^	540	530^a^
	Skopintsev monomer^b^	505	
	Kato monomer^c^	416	
M_like_-state	Based on XRD O-state	452	400^d^
O-state	XRD^a^	606	602^a^
	XFEL^b^	596	

a See ref. (11).

b See ref. (15).

c See ref. (4).

d See ref. (3).

The calculated absorption wavelength of 416 nm for the Kato monomer structure (Table 2) is ∼100 nm shorter than the experimentally measured wavelength in the ground state, mainly because the proton is delocalized over the Schiff base and Asp116 moiety along the low-barrier H-bond (Fig. 3, *A*) due to the absence of the H-bond donations from Ser70 and a cluster of water molecules (Fig. 1*D* and Table 1).

**Figure 5:**
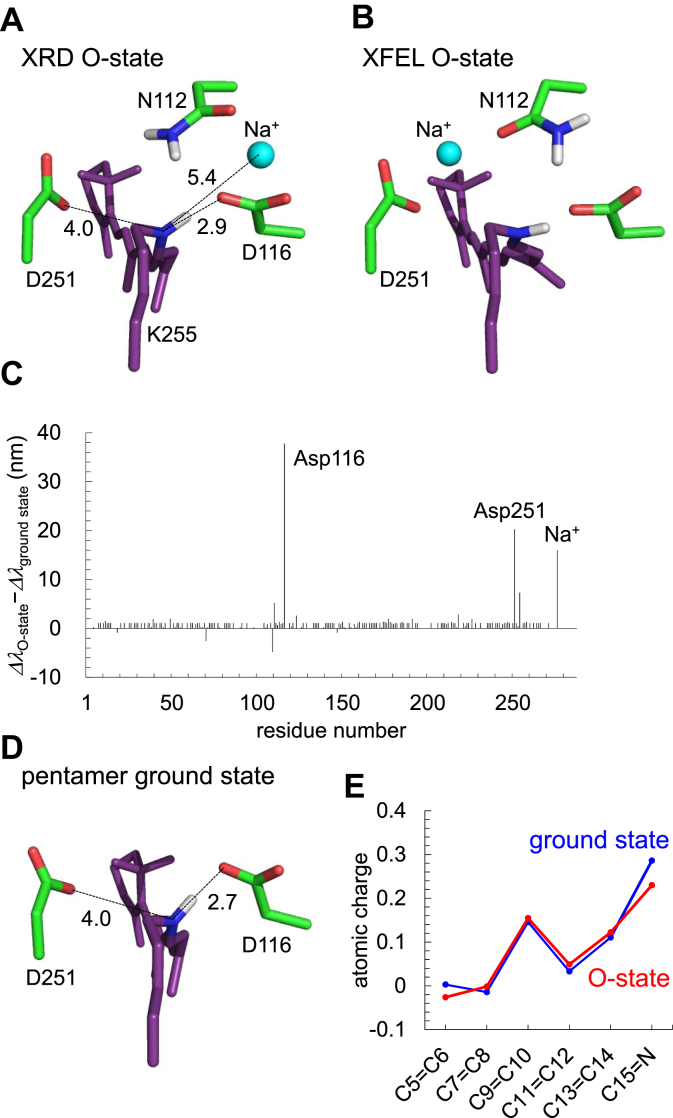
**XRD and XFEL O-state structures.***A–B*, positions of counterion groups. *A*, XRD O-state structure. *B*, XFEL O-state structure. Distances between the N atom of the Schiff base and counterions/Na^+^ are shown in Å. *C*, differences in the electrostatic contributions of side chains/Na^+^ to the absorption wavelength between XRD O-state (Δ*λ*_O-state_) and pentamer ground state (Δ*λ*_ground state_), Δ*λ*_O-state_ − Δ*λ*_ground state_. *D*, positions of counterion residues (Asp116 and Asp251) in the pentamer ground state structure. Distances between the N atom of the Schiff base and counterions are shown in Å. *E*, the S0 state charges of the retinal Schiff base calculated for the pentamer ground and XRD O-state structures.

*O-state*. The calculated absorption wavelength is 606 nm (Table 2) in the XRD O-state structure reported by Kovalev *et al*. (11) (Fig. 5*A*), which is consistent with the experimentally measured wavelength of 602 nm (11). TR-SFX at XFEL by Skopintsev *et al*. suggested that the Na^+^-binding site is formed by Asn112 and Asp251 in the O-state (15) (Fig. 5*B*), in contrast to that in the XRD O-state structure (11) (Fig. 5*A*). A similar site was also proposed by MD simulations (20). The absorption wavelength calculated using the XFEL O-state structure (obtained at 1 ms) (15) is 596 nm (Table 2), which is also consistent with the experimentally measured value (602 nm (11)). These results suggest that both the XRD and XFEL structures are functionally relevant conformations because the experimentally measured absorption wavelength can be reproduced only when the geometry of the retinal Schiff base moiety is functionally relevant (12).

#### Factors that differentiate the absorption wavelengths between the ground and O-states

To identify the factors that differentiate the absorption wavelengths between the pentamer ground and XRD O-states, the electrostatic contribution of each group to the absorption wavelength of the retinal Schiff base was analyzed (Fig. 5*C*). In the XRD O-state structure, Na^+^ near the Schiff base contributes to an increase in the absorption wavelength of 16 nm. Notably, Asp116 and Asp251 (counterion residues) are responsible for the significant difference in the absorption wavelengths (38 and 20 nm, respectively) between the two states (Fig. 5*C*). Both the ground (Fig. 5*D*) and O-state (Fig. 5*A*) structures maintain Asp116 and Asp251 at identical positions with respect to the retinal Schiff base (∼2.8 Å and ∼4.0 Å, respectively). However, the present quantum chemical calculations indicate that in the S0 state, the C=N site of the retinal Schiff base is more positively charged in the pentamer ground state structure than in the O-state structure (Fig. 5*E*). Meanwhile, retinal Schiff base is less distorted in the C13=C14 region in the pentamer ground state structure than in the O-state structure (Table S1). Note that the Schiff base (C=N) region is more positively charged as the C13=C14 region is plainer (Fig. S2). Thus, negatively charged Asp116 and Asp251 stabilize the S0 state in the ground state structure more effectively than in the O-state structure. This could explain why Asp116 and Asp251 are responsible for the difference in the absorption wavelengths between the two states. It seems likely that charged groups near the retinal Schiff base (*i.e.*, Asp116, Asp251, and Na^+^) predominantly determine the absorption wavelength in KR2, as demonstrated for 13 microbial rhodopsins (12).

## Discussion

Asp116 is considered to be deprotonated in the ground state (4); the present results indicate that (i) the H-bond donation from Ser70 to Asp116 and (ii) the formation of a cluster of water molecules near Asp116 are essential components for Schiff base protonation in the ground state (Table 1). The Schiff base is protonated in the pentamer and Skopintsev monomer structures (Fig. 3*A*), whereas the proton is not localized at the Schiff base moiety in the Kato monomer structure (Fig. 3*A*). Among the three ground state structures, Ser70 does not donate an H-bond to Asp116 only in the Kato monomer structure (Fig. 1, *B*–*D*), which destabilizes the Asp116 ionized state and causes the Schiff base proton to migrate toward Asp116.

**Figure 6:**
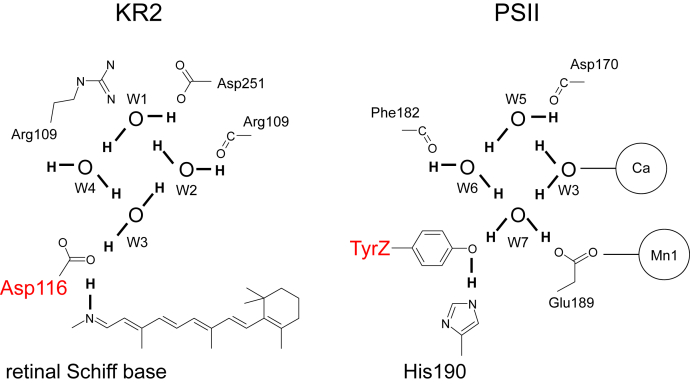
**H-Bond network of the diamond-shaped water cluster near Asp116 in KR2 (*left panel*) and near TyrZ in PSII (*right panel*).** The orientations of the H atoms of water molecules are indicated by *thick lines*.

A cluster of water molecules W1–W4 near Asp116 in the pentamer ground state structure resembles that near redox-active D1-Tyr161 and the H-bond partner D1-His190 in the water-oxidizing enzyme photosystem II (PSII) (21, 22) (Fig. 6). In PSII, the water molecules are more ordered because the water cluster is fixed by the highly polarized oxygen-evolving complex, the Mn_4_CaO_5_ cluster. Thus, one of the water molecules (W7) can donate a stable H-bond to TyrZ and decrease p*K*_a_ (TyrZ) to the level of p*K*_a_ (D1-His190), leading to the formation of a low-barrier H-bond (Fig. 6). In KR2, one of the water molecules (W3) can donate a stable H-bond to Asp116 (Figs. 1*B* and 6), leading to Asp116 ionization and Schiff base protonation (Fig. 3*A*).

In KR2, the Schiff base is deprotonated only in the M state (17). The M_like_-state structure suggests that the Schiff base can be deprotonated by the absence of W2–W4 and the absence of the Ser70 H-bond donation to Asp116 (Fig. 4). The experimentally measured absorption wavelength in the M-state is significantly short (∼400 nm (3)) with respect to the ground (530 nm (11)) and O-states (602 nm (11)). The calculated absorption wavelength is also short (452 nm) for the M_like_-state structure (Table 2), which in turn suggests that the ∼150 nm longer absorption wavelength for the O-state is predominantly due to the incorporation of Na^+^.

**Figure 7:**
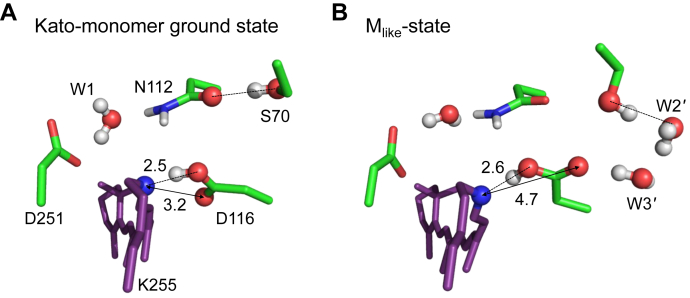
**Schiff base moiety in the QM/MM-optimized (*A*) Kato monomer ground state and (*B*) M**_**like**_**-state structures.** Distances of N_Lys255_…O_Asp116_ are shown in Å. *Dotted lines* indicate H-bonds.

Intriguingly, the absorption wavelength of 416 nm calculated for the Kato monomer ground state structure is closer to the experimentally measured absorption wavelength for the M-state (400 nm) than that calculated for the M_like_-state structure (452 nm, Table 2). This is because Asp116 interacts more strongly with the Schiff base in the Kato monomer ground state structure (N_Lys255_…O_Asp116_ = 2.5 Å and 3.2 Å, Fig. 7) than in the M_like_-state structure ((N_Lys255_…O_Asp116_ = 2.6 Å and 4.7 Å, Fig. 7). In addition, the Schiff base can be deprotonated in both the Kato monomer ground- and M_like_-state structures (Figs. 3*A* and 4*B*), which is characteristic of the M-state (17). Indeed, the Kato monomer ground state structure was originally crystalized at pH 4.0 (4), where Asp116 is more likely to be protonated. Based on the absorption wavelength and the protonation state of the Schiff base, it seems possible that the Kato monomer ground state is more likely to represent the M-state than the ground state.

**Figure 8:**
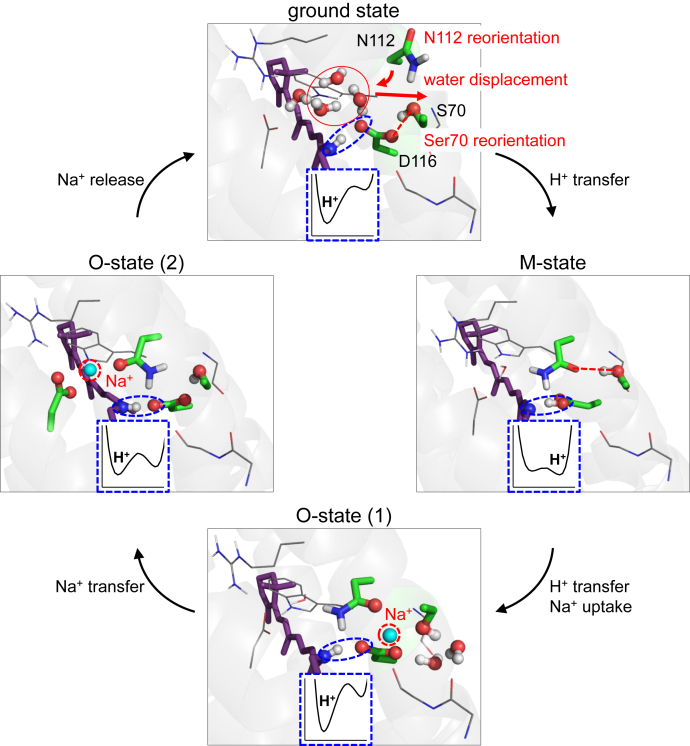
**H**^**+**^**relocation and Na**^**+**^**-pumping mechanism of KR2**.

Based on the observations presented here, we are able to propose a Na^+^-pumping mechanism for KR2 (Fig. 8). In the ground state, Ser70 donates an H-bond to Asp116. The water cluster also donates a stable H-bond to Asp116, as suggested by the QM/MM-optimized pentamer structure (Fig. 1*B*). Thus, the ionized state of Asp116 and the protonated state of the Schiff base are stabilized in the ground state. In the M-state, the H-bond donation to ionized Asp116 from the Ser70 side chain is absent (Fig. 7). In addition, the loss of a cluster of water molecules, which also donates a stable H-bond to ionized Asp116 in the ground state, destabilizes the Asp116 ionization, leading to Schiff base deprotonation (Fig. 4*B*). The link between the loss of the water cluster near Asp116 and the loss of the positive charge of the Schiff base resembles the loss of the water cluster molecules near TyrZ…D1-His190 upon depletion of Ca^2+^ from the Mn_4_CaO_5_ cluster in PSII (23, 24). It seems likely that the retinal Schiff base photoisomerization ultimately induces Asp116 protonation *via* the displacement of water molecules upon M-state formation.

The deprotonation of the Schiff base allows Na^+^ to approach the uncharged Schiff base moiety in the M state. Once Na^+^ is incorporated into the Schiff base moiety upon the O-state formation, the release of the proton from protonated Asp116 toward the Schiff base and the formation of the salt bridge between ionized Asp116 and Na^+^ can be energetically advantageous (Fig. 2*A*). In the O-state, Na^+^ binds initially at Asp116 (*i.e.*, XRD O-state structure, Fig. 2*A*) and subsequently at Asn112 and Asp251 (*i.e.*, XFEL O-state structure, Fig. 2*B*), as suggested in metadynamics simulations (11). Because the calculated absorption wavelengths for the XRD and XFEL O-state structures (606 nm and 596 nm, Table 2) reproduce the experimentally measured wavelength of 602 nm (11) irrespective of the difference in the Na^+^-binding site, it seems likely that both of them represent functionally relevant O-state structures.

## Conclusions

The H-bond donations to Asp116 from the Ser70 side chain and water cluster stabilize the ionized state of Asp116, which leads to p*K*_a_(Schiff base) > p*K*_a_(Asp116) (Fig. 3). The pentamer ground state structure corresponds to the functionally relevant ground state, as the calculated absorption wavelength (540 nm, Table 2) also reproduces the experimentally measured wavelength (530 nm (11)).

To the best of our knowledge, the M-state structure has not yet been reported. The Schiff base is deprotonated only in the M state (17). The Schiff base can be deprotonated due to p*K*_a_(Schiff base) ≈ p*K*_a_(Asp116) in the Kato monomer ground state structure (4) (Fig. 3). In addition, the absorption wavelength calculated for the Kato monomer ground state structure (416 nm, Table 2) reproduces the experimentally measured absorption wavelength (400 nm (3)). The reason for the shortest absorption wavelength is that the two O atoms of Asp116 interact strongly with the Schiff base in the Kato monomer ground state structure, which may hold true for the M-state. The incorporation of Na^+^ into the Schiff base moiety is predominantly responsible for the significant increase of 200 nm in the absorption wavelength upon the M- to O-state transition, as the calculated absorption wavelengths for both the O-state XRD (11) and XFEL (15) structures are identical (∼600 nm, Table 2) and are consistent with the experimentally measured wavelength (11). These results provide a key to understanding how Na^+^ can pass through the positively charged Schiff base moiety in KR2 (Fig. 8).

## Experimental procedures

### Coordinates and atomic partial charges

The atomic coordinates of KR2 were taken from the X-ray structures: pentamer (PDB code 6YC3 (11)), Skopintsev monomer (PDB code 6TK6 (15)), and Kato monomer (PDB code 3X3C (4)) structures for the ground state, and the XRD (PDB code 6XYT (11)) and XFEL (PDB code 6TK2 (15)) structures for the O-state. The monomer unit “A” was used for the pentamer structures. All crystal water molecules and ions were included explicitly in calculations if not otherwise specified. During the optimization of hydrogen atom positions with CHARMM (25), the positions of all heavy atoms were fixed, and all titratable groups (*e.g.*, acidic and basic groups) were ionized. The Schiff base was considered to be protonated. Atomic partial charges of the amino acids and retinal were obtained from the CHARMM22 (26) parameter set.

### Protonation pattern

The computation of the protonation pattern was based on the electrostatic continuum model, solving the linear Poisson–Boltzmann equation with the MEAD program (27). The difference in electrostatic energy between the two protonation states, protonated and deprotonated, in a reference model system was calculated using a known experimentally measured p*K*_a_ value (*e.g.*, 4.0 for Asp (28)). The difference in the p*K*_a_ value of the protein relative to the reference system was added to the known reference p*K*_a_ value. The experimentally measured p*K*_a_ values employed as references were 12.0 for Arg, 4.0 for Asp, 9.5 for Cys, 4.4 for Glu, 10.4 for Lys, 9.6 for Tyr (28), and 7.0 and 6.6 for the N_ε_ and N_δ_ atoms of His, respectively (29, 30, 31). All other titratable sites were fully equilibrated to the protonation state of the target site during titration. The dielectric constants were set to 4 inside the protein and 80 for water. All water molecules were considered implicitly. All computations were performed at 300 K, pH 7.0, and with an ionic strength of 100 mM. The linear Poisson–Boltzmann equation was solved using a three-step grid-focusing procedure at resolutions of 2.5, 1.0, and 0.3 Å. The ensemble of the protonation patterns was sampled using the Monte Carlo (MC) method with the Karlsberg program (32). The MC sampling yielded the probabilities [protonated] and [deprotonated] of the two protonation states of the molecule.

### QM/MM calculations

The geometry was optimized using a QM/MM approach. The restricted density functional theory (DFT) method was employed with the B3LYP functional and LACVP∗ basis sets using the QSite (33) program. The QM region was defined as the retinal Schiff base (including Lys255), side chains of Ser70, Arg109, Asn112, Trp113, Asp116, Tyr218, Asp251, and Ser254, and water molecules and Na^+^ near the Schiff base. All atomic coordinates were fully relaxed in the QM region, and the protonation pattern of the titratable residues was implemented in the atomic partial charges of the corresponding MM region. In the MM region, the positions of the H atoms were optimized using the OPLS2005 force field (34), while the positions of the heavy atoms were fixed. See Fig. S3 for the QM/MM-optimized geometry for the pentamer structure.

To obtain the potential energy profiles of the H-bonds (Figs. 3 and 4*B*), the QM/MM optimized geometry was used as the initial geometry. The H atom under investigation was moved from the H-bond donor atom (N_donor_) toward the acceptor atom (O_acceptor_) by 0.05 Å, after which the geometry was optimized by constraining the N_donor_–H and H–O_acceptor_ distances, and the energy was calculated. This procedure was repeated until the H atom reached the O_acceptor_ atom. All atomic coordinates were fully relaxed in the QM region, whereas only the H atom positions were optimized in the MM region.

The absorption energy of microbial rhodopsins is highly correlated with the energy difference between the highest occupied molecular orbital (HOMO) and lowest unoccupied molecular orbital (LUMO) of the retinal Schiff base (Δ*E*_HOMO-LUMO_) (12, 35). To calculate the absorption energies and the corresponding wavelengths, the QM region was redefined to only include the retinal Schiff base, and the energy levels of the HOMO and LUMO were calculated. The absorption energy (*E*_abs_ in eV) was calculated using the following equation (obtained for 13 microbial rhodopsins; coefficient of determination *R*^2^ = 0.995) (12):

(1) *E*_abs_ = 1.360 Δ*E*_HOMO-LUMO_ – 1.018

A QM/MM approach with the polarizable continuum model (PCM) method with a dielectric constant of 78 for the bulk region, in which electrostatic and steric effects created by a protein environment were explicitly considered in the presence of bulk water, was employed. Here, the polarizable amber-02 force field (36) was applied to the MM region, where the induced dipoles of the MM atoms were considered to reproduce the dielectric screening (*i.e.*, polarizable QM/MM/PCM (37)). In the PCM method, the polarization points were placed on spheres with a radius of 2.8 Å from the center of each atom to model possible water molecules in the cavity. Radii of 2.8–3.0 Å from each atom center and the dielectric constant value of ∼80 are likely to be optimal to reproduce the excitation energetics, as evaluated for the polarizable QM/MM/PCM approach (37). The DFT method with the B3LYP functional and 6-31G∗ basis sets was employed using the GAMESS program (38). The electrostatic contribution of the side chain in the MM region to the absorption wavelength of the retinal Schiff base was obtained from the shift in the HOMO-LUMO energy gap upon removal of the atomic charges of the focusing side chain.

To obtain the charge distribution of the retinal Schiff base for the S0 (ground) state, the restrained electrostatic potential charges (39) of the retinal Schiff base were calculated in the absence of the protein environment. We employed the DFT method with the B3LYP functional and 6-31G∗ basis sets using the Gaussian 16 program (40).

## Data availability

All of the data supporting the findings of this study are available within the paper and the supporting information.

## Supporting information

This article contains supporting information.

## Conflicts of interest

The authors declare that they have no conflicts of interest with the contents of this article.

## Supporting information

Figures, Tables and References
