# Supplementary material for: Identification of intermediate conformations in the photocycle of the light-driven sodium-pumping rhodopsin KR2
Source: J Biol Chem. 2021 Feb 24;296:100459. doi: 10.1016/j.jbc.2021.100459 (PMC8039564; doi:10.1016/j.jbc.2021.100459)
Supplement: Figures, Tables and References [file mmc1.docx]

**Supporting Information**

Identification of intermediate conformations in the photocycle of the light-driven sodium-pumping rhodopsin KR2

Masaki Tsujimura ^1*^ and Hiroshi Ishikita ^1,2^*

1) Department of Applied Chemistry, The University of Tokyo, 7-3-1 Hongo, Bunkyo-ku, Tokyo 113-8654, Japan

2) Research Center for Advanced Science and Technology, The University of Tokyo, 4-6-1 Komaba, Meguro-ku, Tokyo 153-8904, Japan

**Figure S1.** Two possible H-bond patterns of a cluster of four water molecules (W1­–W4) in the pentamer structure. Potential energy profiles of the H-bond between the Schiff base and Asp116 and the corresponding H-bond patterns 1 (discussed in the main text) and 2. It should be noted that p*K*_a_(Schiff base) > p*K*_a_(Asp116) in the two cases.

Figure S2. (a) Correlation between the atomic charges (RESP charges (1)) of the C﻿=N region and the C12–C13=C14–C15 dihedral angle in S0. The initial coordinates of the intermediate structures were obtained interpolating the atomic coordinates of the pentamer ground- and XRD O-state structures linearly. The initial geometry was optimized with the B3LYP functional and LACVP* basis sets, constraining the dihedral angles. For geometry optimization, DFT calculations were performed using the Jaguar program (2). (b) Chemical structure of retinal Schiff base.

**Table S1.** Dihedral angles of the retinal Schiff base in the QM/MM-optimized pentamer ground- and XRD O-state structures (degree).

|  | **Pentamer**  **ground state** ^a^ | **XRD**  **O-state** ^a^ | **Difference** |
| --- | --- | --- | --- |
| C5=C6–C7=C8 | −176.7 | −178.5 | 1.8 |
| C6–C7=C8–C9 | 173.7 | 177.8 | 4.1 |
| C7=C8–C9=C10 | 174.6 | 170.8 | 3.8 |
| C8–C9=C10–C11 | 172.8 | 175.0 | 2.2 |
| C9=C10–C11=C12 | −174.0 | −175.9 | 1.9 |
| C10–C11=C12–C13 | 165.9 | 163.6 | 2.3 |
| C11=C12–C13=C14 | −171.9 | −172.6 | 0.7 |
| C12–C13=C14–C15 | 153.2 | 140.0 | 13.2 |
| C13=C14–C15=N | −176.4 | −171.5 | 4.9 |
| C14–C15=N–CE | 157.7 | 156.3 | 1.4 |

^a^ See ref. (3).

**Figure S3.** QM/MM-optimized geometry (color) and the original crystal structure (gray) for the pentamer structure.

**REFERENCES**

1. Bayly, C. I., Cieplak, P., Cornell, W. D., and Kollman, P. A. (1993) A well-behaved electrostatic potential based method using charge restraints for deriving atomic charges: the RESP model. *The Journal of Physical Chemistry* **97**, 10269-10280

2. Jaguar. (2011). version 7.9, Schrödinger, LLC, New York, NY

3. Kovalev, K., Astashkin, R., Gushchin, I., Orekhov, P., Volkov, D., Zinovev, E., Marin, E., Rulev, M., Alekseev, A., Royant, A., Carpentier, P., Vaganova, S., Zabelskii, D., Baeken, C., Sergeev, I., Balandin, T., Bourenkov, G., Carpena, X., Boer, R., Maliar, N., Borshchevskiy, V., Büldt, G., Bamberg, E., and Gordeliy, V. (2020) Molecular mechanism of light-driven sodium pumping. *Nature Communications* **11**, 2137
